# Alterations in magnitude and spatial distribution of erector spinae muscle activity in cyclists with a recent history of low back pain

**DOI:** 10.1007/s00421-024-05628-7

**Published:** 2024-10-04

**Authors:** Alessandro Sampieri, Giuseppe Marcolin, Federico Gennaro, Emanuele Magistrelli, Alessandro Del Vecchio, Tatiana Moro, Antonio Paoli, Andrea Casolo

**Affiliations:** 1 Department of Biomedical Sciences, University of Padua, Via Marzolo 3, 35131 Padua, Italy; 2 Brain, Mind and Computer Science Doctoral Program, University of Padua, Padua, Italy; 3 Department of Artificial Intelligence in Biomedical Engineering, Friedrich-Alexander University, Erlangen-Nuremberg, Germany

**Keywords:** Low back pain, High-density surface EMG, Erector spinae, Cycling

## Abstract

**Purpose:**

While cycling offers several health benefits, repetitive loading and maintenance of static postures for prolonged periods expose cyclists to low back pain (LBP). Despite high LBP prevalence in cyclists, underlying pathomechanics and specific lumbar region muscle activation patterns during cycling are unclear. Here, we compared lumbar erector spinae (ES) muscles activation and spatial distribution activity in cyclists with and without recent LBP history.

**Methods:**

Ten cyclists with recent LBP history (LBPG; Oswestry Disability Index score ~ 17.8%) and 11 healthy cyclists (CG) were recruited. After assessing the Functional Threshold Power (FTP), participants underwent an incremental cycling test with 4 × 3 min steps at 70%, 80%, 90%, and 100% of their FTP. High-density surface electromyography (HDsEMG) signals were recorded from both lumbar ES using two 64-channel grids. Information about ES activation levels (root-mean-square, RMS), degree of homogeneity (entropy), and cranio-caudal displacement of muscle activity (Y-axis coordinate of the barycenter of RMS maps) was extracted from each grid separately and then grand-averaged across both grids.

**Results:**

Repeated-measure 2-way ANOVAs showed a significant intensity by group interaction for RMS amplitude (*p* = 0.003), entropy (*p* = 0.038), and Y-bar displacement (*p* = 0.033). LBPG increased RMS amplitude between 70–100% (+ 19%, *p* = 0.010) and 80–100% FTP (+ 21%, *p* = 0.004) and decreased entropy between 70–100% FTP (− 8.4%, *p* = 0.003) and 80–100% FTP (− 8.5%, *p* = 0.002). Between-group differences emerged only at 100% FTP (+ 9.6%, *p* = 0.049) for RMS amplitude.

**Conclusion:**

Our findings suggest that cyclists with recent LBP history exhibit higher ES muscles activation and less homogeneous activity compared to healthy controls, suggesting potential inefficient muscle recruitment strategy.

**Trial registration number:**

HEC-DSB/09-2023.

## Introduction

Cycling is one of the most practiced forms of physical activity worldwide for its recognized health benefits (Oja et al. 2011) and low-impact nature due to the reduced stress applied on the joints. However, repetitive loading and maintenance of flexion of the hips and spine for prolonged periods to ensure optimal aerodynamics (Kyle 1994) expose cyclists to a broad range of non-traumatic musculoskeletal complications, symptoms, or overload injuries (Marsden and Schwellnus 2010).

Low back pain (LBP), defined as perceived discomfort between the twelfth rib and the lower folds of the gluteal region with or without leg pain (Krismer and van Tulder 2007), has emerged as one of the most common musculoskeletal complications that affect road cyclists. Indeed, previous studies report a yearly prevalence rate of LBP approximately ranging from 30 to 58% among road cyclists of all experiences (Clarsen et al. 2010; Battista et al. 2021).

Despite cyclists’ vulnerability to LBP has been studied extensively, the precise pathomechanical mechanisms associated with the onset and development of LBP remain largely unknown (Marsden and Schwellnus 2010). Additionally, despite limited existing evidence, specific kinematic mechanism and motor control patterns in cyclists affected LBP are poorly understood. Previous studies showed that cyclists with LBP exhibit an increased lower lumbar spinae rotation and inclination (Burnett et al. 2004) or greater lower lumbar flexion (Van Hoof et al. 2012), which in turn may contribute to increased pain or discomfort. Conversely, a more recent study showed no differences in lower lumbar spinae kinematics in cyclists suffering LBP compared to healthy controls despite observing a lower thoracic spine flexion (i.e., a more upright position) during cycling (Marineau Belanger et al. 2022).

In addition, preliminary research suggests that changes in spine kinematics in LBP cyclists may be accompanied by maladaptive changes in motor control strategies and paraspinal muscle activation patterns. However, at present, only a few studies have documented lumbar spine muscles activation in LBP cyclists, yielding contradictory results. For instance, cyclists with LBP showed a greater asymmetry in the superficial lumbar multifidus before and after prolonged cycling effort in cyclists with LBP (Burnett et al. 2004). Other studies reported increased fatiguability of erector spinae (ES) muscles in LBP cyclists compared to controls (Srinivasan and Balasubramanian 2006), or even no manifestation of myoelectrical fatigue in that specific muscle group (Balasubramanian and Srinivasan 2009). A more recent study (Marineau Belanger et al. 2022) found similar levels of ES muscle activity throughout a 60-min moderate cycling effort between LBP and healthy cyclists. However, differences in cycling protocols and myoelectrical variable analyses hinder, at present, a comprehensive understanding of motor control strategies and muscle activation patterns in cyclists with LBP.

Additionally, all these previous studies employed the traditional bipolar surface electromyography (EMG), a technique characterized by poor selectivity, considerable variability, and low reliability in discerning differences in activation patterns within specific muscle regions (Merletti et al. 2010; Falla and Gallina 2020). These limitations can be addressed by means of multichannel bi-dimensional recording systems, specifically high-density surface EMG (HDsEMG). HDsEMG allows for assessing not only global EMG amplitude (i.e., an estimated degree of muscle excitation) but also regional activation alterations within muscles and the spatio-temporal distribution of muscle activity (Falla and Gallina 2020).

Recently, the implementation of HDsEMG has become widespread for studying muscle activation and distribution among individuals suffering from LBP. In this respect, mounting evidence suggests variations in the overall activity and uniformity of lumbar ES muscle activation in people with LBP compared to healthy controls. For instance, individuals with LBP displayed lower levels of motor variability in the lumbar ES muscles (i.e., reduced spatial redistribution of muscle activity) during sustained back extension (Abboud et al. 2014; Sanderson et al. 2019b) or repetitive lifting tasks (Falla et al. 2014). Some studies reported a lower degree of muscle activity during a singular lifting task (Sanderson et al. 2019a) or higher EMG amplitude during repetitive lifting tasks (Falla et al. 2014). Conversely, others failed to detect any difference in muscle activity during dynamic fatiguing tasks (Arvanitidis et al. 2021), isometric lumbar extension (Arvanitidis et al. 2022), and trunk extension–flexion contractions (Arvanitidis et al. 2024).

Investigating ES muscle activity in sports with a high incidence of LBP is still in its fledging state. To the best of our knowledge, a systematic evaluation of the spatial distribution of ES muscle activity assessed through HDsEMG has been performed only in rowers (Martinez-Valdes et al. 2019). The authors showed that rowers with a recent history of LBP had greater muscle activation and an altered spatial distribution of ES muscles as the load increased, compared to healthy controls (Martinez-Valdes et al. 2019). However, no previous studies have measured lumbar region muscle activation patterns in cyclists, and particularly in cyclists with LBP. Therefore, we aimed to investigate the activation and the spatial distribution of lumbar ES muscles activity in cyclists with and without a recent history of LBP during an incremental cycling test. We hypothesized that cyclists with LBP would exhibit an altered magnitude and spatial distribution of the ES muscles activity compared to healthy controls. This, in turn, might indicate an inefficient pattern of ES muscles recruitment and activation during a cycling task.

## Methods

### Participants

Eleven cyclists with recent history of LBP (LBPG) and 13 healthy cyclists (CG) were recruited from different local cycling clubs and volunteered to participate in this cross-sectional study. To be included in the study, participants had to be aged between 18 and 65 yr., have a body mass index (BMI) less than 30 kg·m^−2^; and pedal at least 4000 km annually for more than two consecutive years. Exclusion criteria were (1) acute traumatic body injury or surgery and (2) history of cardiovascular and respiratory diseases. Specific inclusion criteria for LBPG were (1) non-specific low back pain episodes within the last six months but symptom-free during the last 6 weeks and (2) no pharmacologic interventions for the relief of LBP in the last six months prior to the test. Non-specific LBP refers to symptomatology without an identifiable etiology linked to pathology or trauma (Krismer and van Tulder 2007) and identified by excluding other spinal disorders. The specific inclusion criterion for the CG was no history of acute or chronic LBP within the last year. Three participants (two from the CG and one from LBPG) were excluded from the analyses because excessive sweating during the cycling test negatively affected EMG signal quality. Thus, results are presented for 21 participants (10 LBPG; 11 CG) (Table 1). Informed written consent was obtained by all participants prior to their involvement in the study. Experimental protocols and procedures were approved by the Internal Review Board of the Department of Biomedical Sciences of the University of Padua (HEC-DSB/09–2023) and conformed to the standards set by the Declaration of Helsinki.

**Table 1:** Participants’ baseline characteristics by group. Data are presented as mean ± SD

	Group	
	LBPG (*n* = 10)	CG (*n* = 11)
Age (yr)	42.2 ± 11.9	37.3 ± 13.1
Gender (*n*)	M = 10	M = 10; F = 1
BMI (kg·m^−2^)	23.4 ± 2.4	22.5 ± 1.4
IPAQ-SF score (MET min·wk^−1^)	6781.0 ± 3480.0	8261.4 ± 3881.0
ODI-I (%)	17.8 ± 10.3	0
CPG (pain intensity score)	33.3 ± 4.4	0
CPG (disability score)	15.3 ± 23.8	0
FTP (W/Body Mass)	3.4 ± 0.7	3.8 ± 1.0

*M* males, *F* females, *BMI* body mass index, *ODI-I* Oswestry Disability Index—Italian Version, *CPG* chronic pain grade questionnaire – Italian Version, *FTP* functional threshold power

### Questionnaires

The participants completed multiple questionnaires to assess their overall health and cycling habits. First, to estimate weekly physical activity levels, participants completed the International Physical Activity Questionnaire (IPAQ-SF (Mannocci et al. 2010), which is known for its acceptable validity and reliability across various populations (Craig et al. 2003). Second, to assess the level of disability related to LBP among the LBPG, we used the Oswestry Disability Index (ODI-I, Italian Ver. 2.1a (Monticone et al. 2009)), which shows high reliability and validity, especially for minor levels of disability (Fairbank and Pynsent 2000). Last, we administrated the validated Italian version of the Chronic Pain Grade (CPG) questionnaire, which assessed both the characteristic pain intensity and the disability related to LBP (Salaffi et al. 2006). This questionnaire classifies respondents into 4 categories: Grade 0 (no pain, no disability); Grade 1 (low disability, low intensity); Grade 2 (low disability, high intensity); Grade 3 (high disability, moderately limiting); and Grade 4 (high disability, severely limiting).

### Study design

Data collection was performed over a five-month period at the Nutrition and Exercise Physiology Laboratory, at the Department of Biomedical Sciences of the University of Padua, Italy. Each participant attended the laboratory for two experimental sessions separated by 7–10 days.

In the first session, participants underwent anthropometric measurements and performed the validated Carmichael Training System (CTS) Field Test (Carmichael and Ruttberg 2012), to determine their Functional Threshold Power (FTP). In the second session, participants performed the incremental cycling test with the concomitant recording of HDsEMG from lumbar ES muscles (see Testing and procedures for details).

In both sessions, participants rode their own road bicycle mounted on a smart trainer device (Elite Direto XR, Padua, Italy). Furthermore, participants were asked not to engage in any strenuous physical exercise and to avoid caffeine or energy drink consumption within 48 and 24 h before each session, respectively.

### Experimental protocols

#### CTS field test

The smart trainer device allowed participants to use their own bikes and replicate their habitual riding posture. Moreover, the device is equipped with a power meter based on an optical sensor technology that measures the torsion of the trainer axis with a ± 1.5% accuracy.

The CTS Field test was used to determine participants’ FTP (i.e., the maximum power (Watt) that a cyclist can sustain over 60 min (Allen and Coggan 2010)). After 10–15 min of warm-up, characterized by an easy-to-moderate pedaling and 3 × 30 s of moderate-to-high progressions, participants completed two 8 min steps at their maximum sustainable intensity. They were instructed to maintain a cadence between 80 and 100 revolutions·min^−1^ (RPM) and push themselves as hard as possible without slowing down or standing up from the saddle for the 8 min “all-out” effort. A 10 min low-intensity cycling period separated the two 8-min steps. During each step, the power output was recorded using the software MyETraining (v. 1.18.3.0; Elite Srl, Padua, Italy). FTP value was calculated by subtracting the 10% from the average mean power outputs achieved during the two 8 min steps (Carmichael and Ruttberg 2012).

#### HDsEMG data collection during the incremental cycling test

**Fig. 1:**
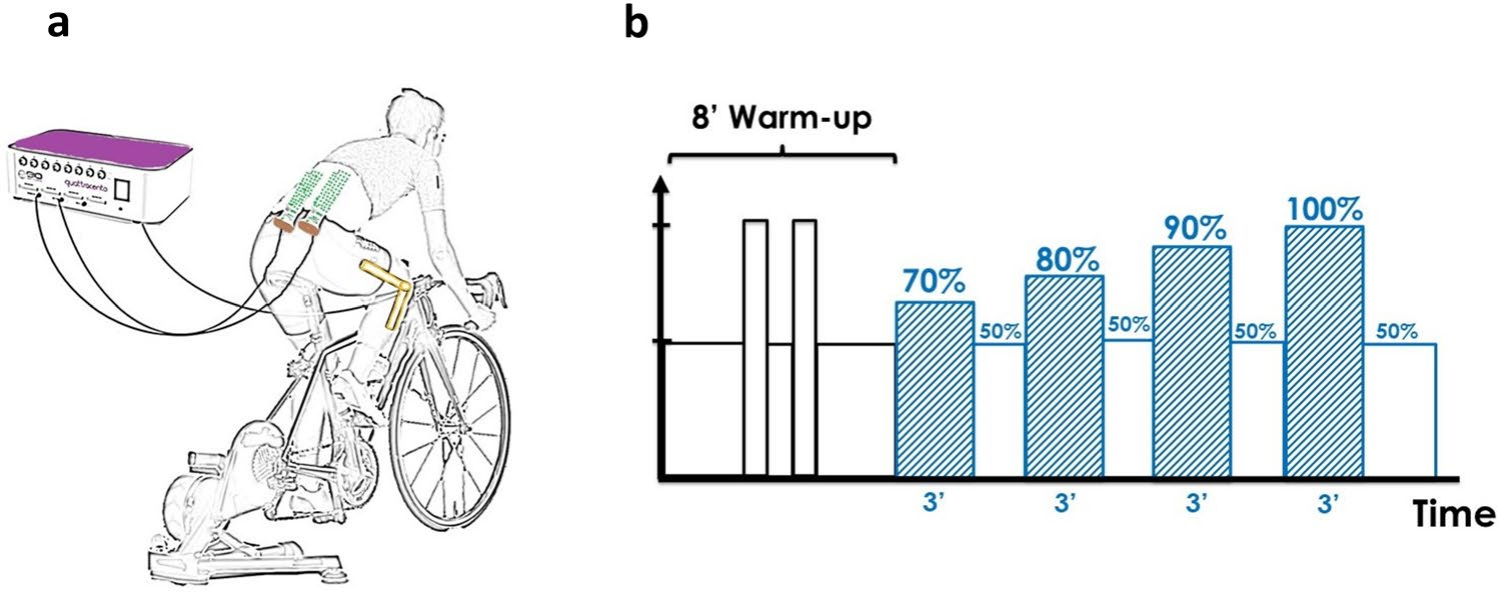
**a** Experimental Set-Up: a participant is seated on his bike mounted on a smart trainer device. Two semi-disposable adhesive grids of electrodes (13 rows × 5 columns, 8 mm inter-electrode distance) positioned over the ES muscles are connected to the multichannel EMG amplifier. An electro-goniometer (EGN) positioned on the right knee was used to characterize the pedal stroke. **b** Experimental protocol: after a standardized warm-up phase, participants performed an incremental cycling test consisting of 4 steps of 3 min interspersed with 2 min of rest at 50% FTP

In the second session, the same experimental setup of session 1 was used with the addition of the HDsEMG electrodes to record the EMG activity and the electro-goniometer (EGN) to identify the pedal strokes (Fig. 1a).

Prior to electrode placement, the skin surface was shaved, lightly scrubbed with abrasive paste (Everi, SPES Medica, Genoa, Italy) to reduce skin impedance, and cleansed with alcohol. Two high-density grids of 64 equally-spaced electrodes (13 rows (10.0 cm) × 5 (3.5 cm) columns, gold-coated, with a 1 mm diameter and 8 mm inter-electrode distance) were prepared before placement with a double-sided adhesive foam layer. Electroconductive paste (AC Cream SPES Medica, Genoa, Italy) was used to fill the holes in correspondence with the electrodes. An experienced operator (kinesiologist) attached the two grids of electrodes bilaterally over the surface of the lumbar ES muscles at a standardized position (i.e., 2 cm lateral to the lumbar spinous processes, starting from L5 level to L3 level approximately) as previously described (Barbero et al. 2012; Martinez-Valdes et al. 2019). This positioning ensures that recordings are made from the lower lumbar fascicles of the ES, specifically targeting the iliocostalis lumborum, pars lumborum and pars thoracis (Sanderson et al. 2019b). Initially, hypafix tape was used to enhance skin–electrode contact. Subsequently, to further minimize grid detachment due to sweating, a cohesive bandage (Phytop, Wuxi Jiangsu, China) was lightly applied around the trunk. This application was carefully adjusted to ensure it did not cause any constriction or discomfort for the cyclists during pedaling. The HDsEMG signals were recorded in monopolar derivation, amplified (× 150), sampled at 2048 Hz, band pass-filtered at source (10–500 Hz), and converted to digital data by a 16-bit A/D multichannel amplifier (EMG-Quattrocento, OT Bioelettronica, Turin, Italy), prior to offline analysis.

A uniaxial EGN was positioned on the right knee, with the fulcrum located proximally to the lateral condyle to collect the knee flex–extension while pedaling. The EGN signal was sampled at 2048 Hz and synchronized with HDsEMG signals by the same multichannel amplifier (EMG-Quattrocento, OT Bioelettronica, Turin, Italy).

After the positioning of the sensors, participants performed a standardized warm-up as follows: 3 min at 50% FTP, 1 min at 100% FTP, 1 min at 50% FTP, 1-min at 100% FTP and 2-min at 50% FTP. Thereafter, they performed 4 steps of 3-min at 70%, 80%, 90%, and 100% FTP, respectively. A 2-min recovery at 50% FTP was performed after each step (Fig. 1b). Participants were instructed to maintain their self-selected cycling pace, allowing them to preserve their habitual pedaling technique during the whole test. Both HDsEMG and EGN signals were recorded continuously throughout the incremental cycling test.

#### Data analysis

**Fig. 2:**
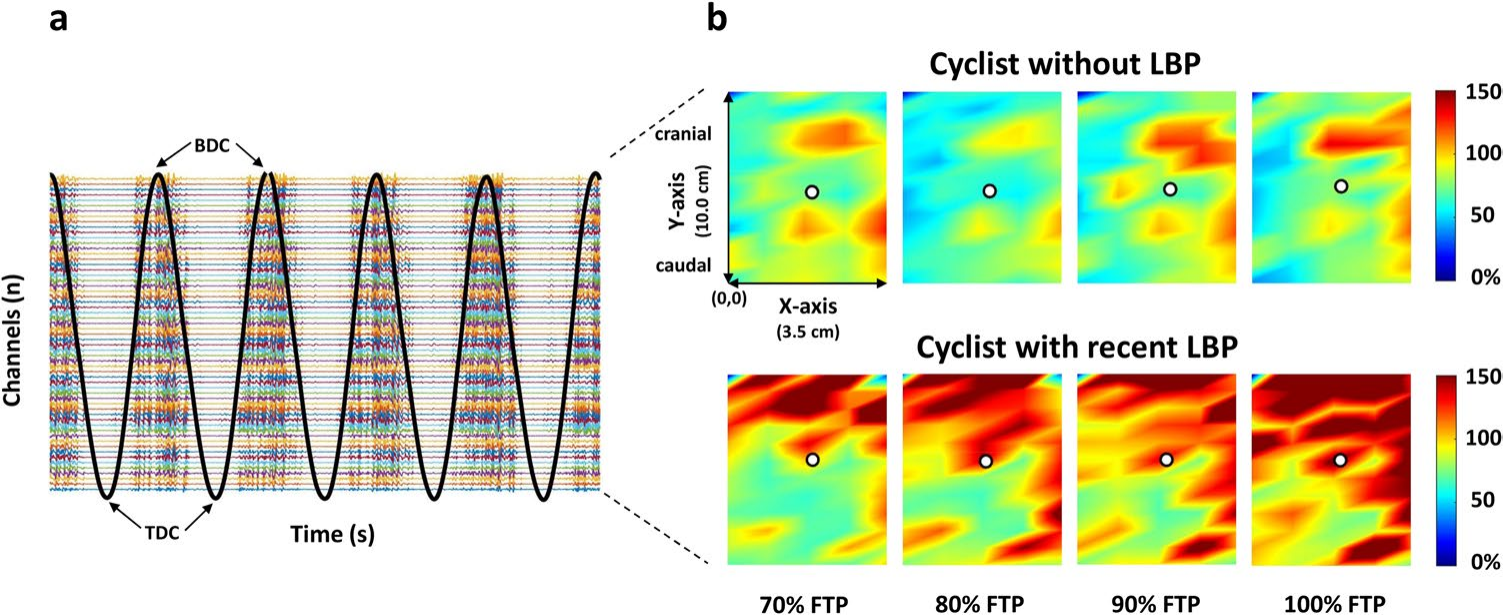
**a** Example of 59 single differential HDsEMG signals recorded from the right erector spinae (ES) muscle for one representative participant during the test. The black line indicates the electro-goniometer (EGN) signal used for the identification of the pedal strokes. TDC and BDC indicate, respectively, the top dead center and the bottom dead center. Each right pedal stroke was defined from one TDC to the subsequent TDC. Note the periodic burst of activation of the right ES for each corresponding pedal stroke; **b** Representative RMS maps of average EMG amplitude recorded from ES of one cyclist without LBP (on the top) and for one cyclist with recent history of LBP (on the bottom) at increasing cycling intensities (% FTP). The RMS maps are normalized for average RMS value at 50% FTP. Note the higher activity of the ES muscle in the LBP cyclist. Areas with dark red correspond to higher RMS amplitude. The white circles indicate the position of the barycenter (Y-bar) of the ES activity

HDsEMG recordings were analyzed offline using Matlab R2022b (Mathworks Inc, Natick, MA, USA) and Fieldtrip toolbox (Oostenveld et al. 2011). First, monopolar HDsEMG recordings were band pass-filtered at 30–400 Hz using a second-order, zero-lag, Butterworth filter. Thereafter, each filtered recording was visually inspected and bad channels defined as those not physiologically plausible raw EMG channels (e.g., extremely noisy or flat) were identified by means of one or more of several toolbox-supported metrics (e.g., kurtosis, variance), and subsequently removed. Channel visual inspection and removal strategy were performed with the Fieldtrip toolbox (Oostenveld et al. 2011) and have been applied in several other electrophysiological studies (Nordin et al. 2020; Shirazi and Huang 2021). Those channels were then reconstructed by averaging the nearest neighboring channels. Afterward, the preprocessed signal underwent single differential computations by subtracting adjacent preprocessed monopolar signals along each column of the grid, yielding 59 bipolar signals. This approach was chosen to extract information about muscle activation aligning with the presumed orientation of the lumbar ES muscle fibers (Mawston and G. Boocock 2015). HDsEMG analysis was performed by considering only the central ~ 2-min of each %FTP step. Within these time windows and for each cycling intensity step, individual pedal strokes were identified from the EGN recordings. Specifically, we identified the top dead center (TDC) with the maximum knee flexion and the bottom dead center (BDC) with the maximum knee extensions. Thus, each right pedal stroke extends from one TDC to the subsequent TDC, while the left pedal stroke from one right BDC to the subsequent right BDC (Fig. 2a). This method served to calculate the mean cadence for each participant at each %FTP.

Subsequently, the root-mean-square amplitude (RMS) was calculated from the 59 bipolar signals for each pedal stroke within each %FTP step. The obtained RMS values were then averaged over the 59 signals and for all pedaling strokes, yielding an average RMS value (RMS_MEAN_) for each %FTP step. To enable the comparison among individuals, the RMS values were normalized to the average RMS value expressed in the first minute at 50% FTP. This normalization against a submaximal contraction is considered preferable for individuals affected by LBP that might face challenges in achieving maximal muscle activation (Ng et al. 2002) and has demonstrated high sensitivity in detecting alterations in muscle activation during cycling (Martinez-Valdes et al. 2016) and rowing (Martinez-Valdes et al. 2019) as the intensity increased. Thereafter, electromyographic activation maps (RMS maps) were extracted for each %FTP step, enabling a visuospatial distribution of ES activity (Fig. 2 b). The RMS maps provide insights into the intensity of muscle activity across different points of the acquisition grid. Furthermore, the modified entropy was computed as previously described (Farina et al. 2008) from the normalized RMS values to characterize the complexity of the EMG signal (i.e., degree of homogeneity or heterogeneity in muscle activation). Specifically, higher values correspond to a more homogeneous distribution of muscle activity and refer to a pattern of activation that is less localized and more equally distributed along the muscles. Conversely, a more heterogeneous signal suggests a more localized and less-uniform muscle activation. Finally, the muscle activity’s barycenter was calculated, yielding an indication of the average location of muscle activity. Particularly, the Y-axis coordinate of each RMS map’s barycenter (Y-bar) was analyzed to assess the cranio-caudal displacement of the barycenter at increasing cycling intensities. All channels were considered for this calculation.

Further statistical inference was performed on the averaged left- and right-side ES muscle activity, resulting in a single value per subject and %FTP step for RMS_MEAN_ modified entropy, and Y-bar coordinates. This decision was made upon the rationale that participants in the LBPG had non-specific LBP (i.e., not localized to a specific side or both sides), and an additional statistical test failed to detect significant differences between left and right RMS_MEAN_, modified entropy, and Y-bar coordinates in LBPG and CG.

### Statistical analysis

An a priori power analysis calculation was performed using the G*Power V.3.1.9.4 software (Heinrich Heine University, Dusseldorf, Germany) to determine the required sample size. RMS_MEAN_ was specified as the primary outcome based on previous studies indicating alteration of RMS amplitude with increased load during specific tasks (Falla et al. 2014; Martinez-Valdes et al. 2019). By setting the α risk at 0.05, the statistical power at 0.8, and the effect size f at 0.35, it has been estimated that 20 participants were required to detect significant differences in the RMS_MEAN_. To account for potential loss of data due to signal quality or participant withdrawal, a total of 24 participants were recruited.

The normality of data distribution and the sphericity hypothesis were tested with the Shapiro–Wilk and Mauchly tests, respectively. When the sphericity assumption was violated, the Greenhouse–Geisser correction was applied. Moreover, a Levene’s test was used to check for the homogeneity of variances. Baseline anthropometric characteristics, ODI-I, and IPAQ were compared between groups by independent t-tests. For each EMG parameter (RMS_MEAN_, modified entropy, and Y-bar coordinates), interaction and main effects were checked with a 2-way mixed ANOVA for repeated measures with group (LBPG vs. CG) and FTP intensities (70%, 80%, 90%, and 100% FTP) as between and within factors, respectively. Partial eta-squared (η_p_^2^) was calculated to measure the amount of variance of a dependent variable attributable to a given independent variable, considering the influence of the other independent variables present in the model. A η_p_^2^ less than 0.06 indicates a small effect, between 0.07 and 0.14 a medium effect, and greater than 0.14 a large effect (Cohen 1988). If a significant group by FTP intensities interactions was found, the Bonferroni post hoc analysis was run for multiple comparisons. Statistical significance was set at *p* < 0.05. All statistical tests were performed with the software package JASP V. 0.16.4.0 (JASP Team, Amsterdam, the Netherlands).

## Results

### Participants’ characteristics

The two groups were homogeneous in terms of baseline anthropometric characteristics, physical activity habits, and FTP values (Table 1; *p* > 0.05 for all the characteristics). The LBPG reported an average ODI-I score of 17.8 ± 10.3%, which indicates minimal disability (Fairbank and Pynsent 2000) but significantly (*p* < 0.001) higher than CG, as expected. According to the CPG questionnaire, in the LBPG, 2 participants out of 10 were classified as Grade III (high disability, moderately limiting), while the others as Grade I (low disability, low intensity). Furthermore, the pain score assessed with the CPG questionnaire was, on average, 33.3 ± 4.4, while the disability score was 15.3 ± 23.8. All the participants completed the cycling test without reporting pain.

### RMS, modified entropy and Y-bar displacement

**Fig. 3:**
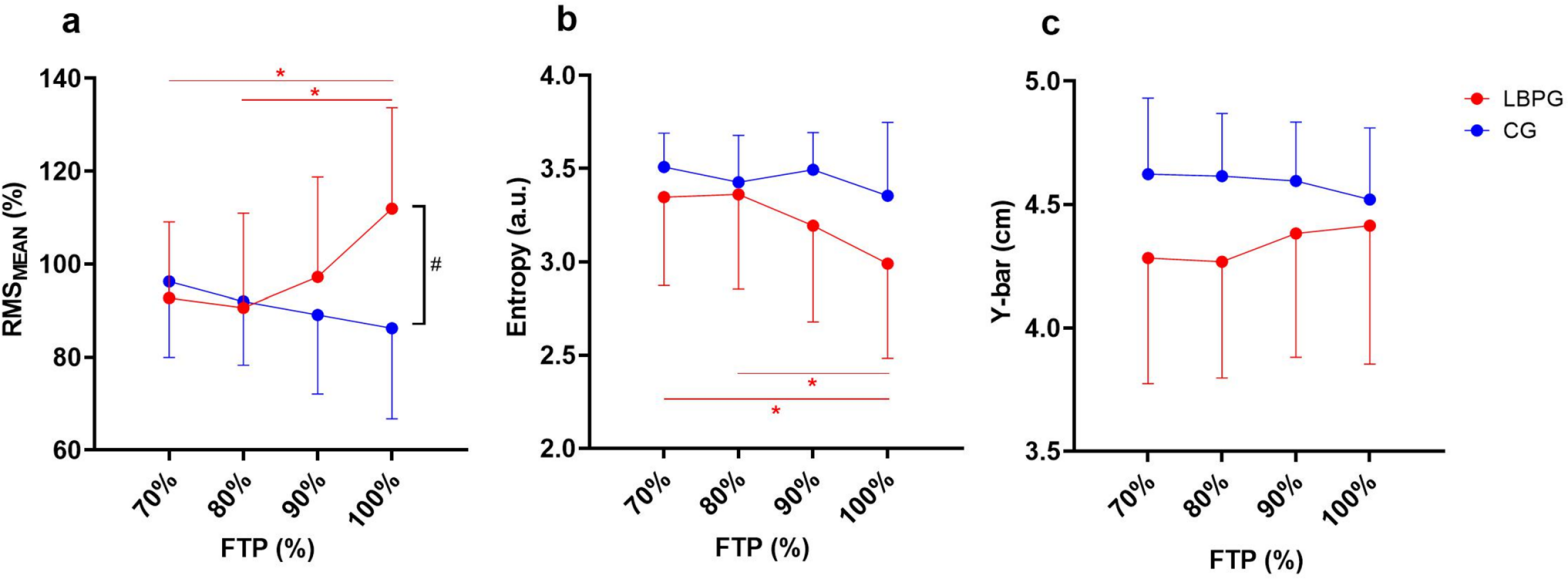
**a** Average normalized RMS amplitude, **b** modified entropy computed from the normalized RMS amplitude, and **c** Y-bar values of ES at each of the four loads of the incremental cycling test (70%, 80%, 90%, 100% FTP). Cyclists with a recent history of low back pain (LBPG) are shown in red, whereas healthy cyclists (CG) are shown in blue; FTP, functional threshold power; *, within group difference (*p* < .05); #, significant (*p* < .05) difference in RMS amplitude between groups at 100% FTP (*p* < .05); A.U., arbitrary units

A significant 2-way interaction effect (load x group) was observed for normalized ES RMS_MEAN_ values (*F* = 7.891, *p* = 0.003, η_p_^2^ = 0.293) and modified entropy (*F* = 3.398, *p* = 0.038, η_p_^2^ = 0.152). Post hoc analyses revealed that LBPG increased their ES RMS_MEAN_ values as the load increased, specifically between 70 and 100% FTP (+ 19.0%, *p* = 0.010) and between 80 and 100% (+ 21.0%, *p* = 0.004), whereas the CG showed no statistically significant differences (Fig. 3a). Furthermore, between groups comparisons showed higher ES RMS_MEAN_ values in LBPG compared to the CG at 100% FTP only (+ 9.6%, *p* = 0.049) (Fig. 3a).

Post hoc analyses for modified entropy comparisons revealed that LBP cyclists had lower entropy values (i.e., increased heterogeneity of activation) as the cycling load increased, particularly between 70 and 100% FTP (-8.4%, *p* = 0.003) and between 80 and 100% FTP (-8.5%, *p* = 0.002), whereas CG cyclists maintained a relatively constant level of entropy throughout the incremental test, as suggested by the absence of significant differences after performing multiple comparisons (Fig. 3b).

Although a significant 2-way interaction effect (load x group) was observed for Y-bar coordinates displacement as well (*F* = 3.745, *p* = 0.033, η_p_^2^ = 0.165), post hoc analysis did not show any within or between-group difference while % FTP increased (Fig. 3c).

## Discussion

The aim of the study was to investigate the level of activation and the spatial distribution of lumbar ES muscles activity in cyclists with a recent history of LBP compared to a cohort of cyclists without LBP. In line with our initial hypothesis, LBPG cyclists exhibited a higher activation of ES muscles and a more heterogeneous distribution of activity as the cycling load increased. Our results suggest a potentially inefficient recruitment strategy of ES muscles in cyclists recently affected by LBP.

Indeed, as the cycling intensity (%FTP) increased, we observed that cyclists with a recent history of LBP required higher activation of ES muscles to achieve the same motor output as the CG cyclists. Conversely, CG maintained a relatively constant level of muscle activation throughout the test. Our results are consistent with previous studies where ES activation increased with the increase of workloads in individuals with chronic non-specific LBP in repetitive lifting tasks (Falla et al. 2014) or in rowers with a recent history of LBP (Martinez-Valdes et al. 2019). However, such differences might not be observed during low-load activities, as highlighted in both LBP patients and healthy individuals  while performing less demanding different tasks (Matheve et al. 2023).

The increment of RMS amplitude in LBPG may result from the increase of the excitatory neural drive to the ES muscles, in turn, associated with an increase in the number of recruited motor units, or an increase of their discharge rate, or a combination of both factors (Carpentier et al. 2001). As suggested previously (Carpentier et al. 2001; Falla et al. 2014), the increase in excitatory drive to muscle may be a strategy to counterbalance the alteration in fiber properties observed in individuals experiencing pain. However, since LBPG did not experience pain during the test, we believe that the greater activation of ES muscles may be the consequence of different trunk kinematics. Indeed, previous studies showed that cyclists affected by LBP experience greater flexion and rotation in the lower lumbar spine compared to healthy cyclists (Burnett et al. 2004; Van Hoof et al. 2012). A second explanation to account for the increased activation of ES muscles may be a dysfunctional neuromotor adaptation rather than a reflexive reaction to pain, considering that the LBPG did not exhibit any painful symptoms during the test.

Our findings of greater ES muscle activity in LBP cyclists compared to healthy participants differed from those of a previous pilot study (Burnett et al. 2004). However, in the mentioned study, cyclists had pain while pedaling and were tested at a constant intensity (i.e., 75% of the maximum heart rate predicted based on their age), which approximately corresponded to 80% FTP according to available training zones (Allen and Coggan 2010). In our study, we adopted an incremental cycling test characterized by stepwise increases in %FTP since research has shown that power output impacts the activation of paravertebral lumbar muscles (Usabiaga et al. 1997; Muyor et al. 2022). Therefore, the adoption of such a paradigm was expected to provide a more in-depth comprehension of potential alterations in both the magnitude of activation and spatial distribution of ES muscles activity at different levels of exertion. Here, since between-group differences appeared only at 100% FTP, we can assume that the alterations in ES muscles level of activation could be particularly evident at the higher pedaling intensities.

The adoption of HDsEMG has further provided indications of changes in the distribution and uniformity of muscle activity associated with LBP. Our results are in agreement with previous reports showing a less-homogeneous ES muscles activity in LBP participants during specific tasks (Sanderson et al. 2019a; Hao et al. 2020). Interestingly, in the present study, LBP cyclists showed lower entropy values as the %FTP intensity increased, which may suggest a more heterogeneous distribution of ES activity as the load increased. This reduction in entropy may be interpreted as a specific neuromuscular control strategy to reorganize the ES muscles’ spatial activity to prevent an overload of muscle regions susceptible to pain. However, further research is warranted to investigate whether an increase in ES muscle activation and a reduction of EMG signal homogeneity may represent contributing factors to or may be the result of LBP onset. Whether this activation pattern is a cause or a consequence of LBP, a less homogeneous activation pattern may decrease the load along the entire ES, leading to increased fatigue of the most activated muscle areas (Arvanitidis et al. 2021) that may alter trunk muscle coordination and spinal stability (Schinkel-Ivy and Drake 2019). This alteration is known to increase the susceptibility to musculoskeletal injuries (Srinivasan and Balasubramanian 2006) and may contribute to the perpetuation or reappearance of pain caused by a less diffuse distribution of ES activation (Falla et al. 2017). Indeed, in the short term, this adaptation strategy may offer protection to muscle regions affected by pain, while over the long term, this activation pattern could lead to increased load and decreased variability (i.e., redistribution of the muscle activation to achieve a motor output) (Hodges and Tucker 2011). Conversely, a more uniform activation within ES muscles during sustained or repetitive tasks may prevent potential musculoskeletal injuries by redistributing the load across the entire ES muscles (Falla and Gallina 2020; Arvanitidis et al. 2021). Therefore, based on the observed findings, we could hypothesize that the ES activation pattern observed in cyclists with LBP places them at a significantly higher risk of experiencing further complications or exacerbating their existing condition.

Concurrently with decreased entropy, the higher effect size (η_p_^2^ = 0.165) in the Y-bar analysis is suggestive of a cranial shift of the center of activity of the RMS map as the load increased although post hoc analysis did not reach the statistical significance. This observation might be attributed to the absence of pain during the test among our cyclists or their mild-to-moderate level of disability (as reported by the ODI-I and CPG Questionnaires). Additionally, the relatively short duration of the test may have influenced these findings, as prior research demonstrated that the displacement of the barycenter increased with longer durations of muscle contraction (Farina et al. 2008). Hence, increasing the duration of each step or the number of %FTP steps might have led to observe significant changes in the position of the barycenter of ES muscle activity. Notably, our findings are consistent with other studies that have observed a cranial shift of ES muscle activity during a isokinetic fatiguing task (Arvanitidis et al. 2021) or singular monoplanar task (Sanderson et al. 2019a) in individuals with LBP. Conversely, our results differ from previous observations in rowers with a recent history of LBP, who displayed a more caudal displacement of the ES activity barycenter as the load increased (Martinez-Valdes et al. 2019), and from non-athletes affected by LBP during lifting tasks, where no significant variations in the distribution of ES muscle activity were observed (Falla et al. 2014). Despite methodological differences across studies that may preclude a direct comparison and interpretation of the results, discrepancies between our results and those of previous studies may indicate the existence of multiple muscle activation patterns that, in turn, may be dependent on the specific task performed or assessed.

There are some limitations in the current study that should be acknowledged. First, we did not assess the trunk kinematics and the activity of the core muscles which may have provided insights into posture and muscular compensation strategies employed while pedaling. Second, due to the relatively similar severity of disability among cyclists with a recent history of LBP, we could not stratify participants based on pain intensity, potentially limiting the generalizability of our results to those with more severe pain or acute episodes of LBP. Last, we did not know the natural history of LBP, which may have been helpful for a more comprehensive interpretation of the results.

To conclude, further studies should investigate whether and how the different activation patterns of the lumbar ES muscles of cyclists with recent history of LBP influence cycling performance. Also, a novel insight may emerge from the implementation of real-time HDsEMG biofeedback, which could assist individuals in adopting different muscle activation behaviors while cycling. This approach has already been recommended for rowers with a recent history of LBP to facilitate more efficient lumbopelvic motion (Martinez-Valdes et al. 2019).

## Conclusion

Our study is the first to provide novel insights into the neuromuscular control mechanisms of ES muscles in cyclists with and without a recent history of LBP. Cyclists with a recent history of LBP showed an over-activation of ES muscle and a sub-optimal strategy to redistribute muscle activity as the cycling load increased, compared to healthy controls. The findings let suppose the presence of altered motor control strategies in cyclists with LBP and may be considered by clinicians, therapists, and coaches to implement and monitor both preventive and treatment strategies for LBP in this population.

## Data Availability

The data that support the findings will be made available by the corresponding author upon reasonable request.

## Abbreviations

ANOVA: Analysis of variance
BDC: Bottom dead center
BMI: Body mass index
CG: Control group
CTS: Carmichael Training System
EGN: Electro-goniometer
EMG: Electromyography
ES: Erector spinae
FTP: Functional threshold power
IPAQ-SF: International physical activity questionnaire short form
HDsEMG: High-density surface electromyography
LBP: Low back pain
LBPG: Low back pain group
ODI-I: Oswestry disability index
RMS: Root-mean-square
RMS_MEAN_: Average RMS value
RPM: Revolutions per minute
TDC: Top dead center
